# Acute serum sodium concentration changes in pediatric patients undergoing cardiopulmonary bypass and the association with postoperative outcomes

**DOI:** 10.1186/s40064-015-1436-2

**Published:** 2015-10-24

**Authors:** Jeong Jin Lee, Young-Soon Kim, Hae Hyuk Jung

**Affiliations:** Department of Anaesthesiology and Pain Medicine, Samsung Medical Center, Sungkyunkwan University School of Medicine, Seoul, South Korea; Department of Anaesthesiology and Pain Medicine, Seoul Woman’s Hospital, Gyeonggi-do, Bucheon, South Korea; Department of Medicine, Kangwon National University Hospital, Kangwon National University School of Medicine, 156 Baekryung-ro Gangwon-do, Chuncheon, 200-722 South Korea

**Keywords:** Cardiopulmonary bypass, Hyponatremia, Hypernatremia, Postoperative complication, Hospital stay

## Abstract

The objective of this study is to investigate the degree of serum sodium changes and its association with patient outcomes in pediatrics undergoing heart surgery with cardiopulmonary bypass (CPB). We reviewed the medical records of 275 pediatric patients who underwent heart surgery with CPB. Prior to CPB, hyponatremia (≤135 mmol/L) was observed in 21 of 275 patients. After initiation of CPB, serum sodium decreased significantly and severe hyponatermia (≤130 mmol/L) subsequently developed in 32 patients. At the end of CPB, however, hypernatremia (≥145 mmol/L) developed in 86 patients. The degree of acute serum sodium change during CPB was not associated with patient outcomes. However, the patients with preoperative hyponatremia and those with hypernatremia at the conclusion of CPB had longer hospital stays and higher postoperative complication rates. Lower serum sodium prior to CPB and higher serum sodium at the end of CPB, along with age and duration of the operation, were independently associated with worse in-hospital outcomes. Acute and transient hyponatremia occurred frequently after initiation of CPB, and then serum sodium immediately increased above preoperative levels at the end of CPB. Caution is required to avoid serum sodium overcorrection on the conclusion of CPB.

## Background

Hyponatremia is a common complication prior to initiating cardiopulmonary bypass (CPB), and is usually secondary to underlying heart disease or medications. Serum sodium concentration may further change during CPB and may be due to a variety of causes, including the administration of large fluid volumes (bypass prime solutions, cardioplegia, and transfusions) (Darling et al. 2000), the use of diuretics, impaired water excretion caused by the release of anti-diuretic hormone (Philbin et al. 1977), and CPB-related natriuretic effect (Sehested et al. 1997).

Sodium disturbances leading to hyponatremia and hypernatremia are common problems in hospital-admitted patients and are associated with poor in-hospital outcomes (Stelfox et al. 2008; Palevsky et al. 1996; Arampatzis et al. 2012). It has been shown that hyponatremia is one of the predictive factors of prognosis and length of hospital stay of patients with congestive heart failure (Mohammed et al. 2010), neurologic disease (Spatenkova et al. 2013), and liver failure (Londono et al. 2006). Conversely, abrupt normalization of hyponatremia can cause more detrimental effects, leading to morbidities such as central pontine myelinolysis (Sterns et al. 1986). However, the epidemiology of sodium disturbances during CPB has not been well defined, and the implication of dysnatremia in patients undergoing cardiac surgery has rarely been demonstrated.

When considering the relatively small extracellular fluid volume, the risk of acute changes in serum sodium may be increased in pediatric patients. The objective of this study is to investigate the degree of changes in serum sodium concentration and its relationship with adverse outcomes in pediatric patients undergoing cardiac surgery with CPB.

## Methods

### Subjects

A total of 275 pediatric patients (≤15 years of age) who underwent surgical repair of ventricular or atrial septal defects under CPB were identified by retrospective review of all CPB cases performed at Samsung Medical Center (Seoul, Korea) from July 2005 to June 2010. During the study period, the total number of cardiac surgery cases under CPB from patients of all ages was 3608 including 1651 surgeries in pediatric patients (≤15 years of age). We only included repairs of ventricular or atrial septal defects to minimize the effects of confounding factors (e.g., the type and duration of operation procedure or the severity of underlying heart disease) on patient outcomes. Patients with a past medical history of chronic hypoxemia (room air saturation <90 %), central nervous system abnormalities, or chromosomal abnormalities were not included.

The study protocol was approved by our institutional review board (Samsung Medical Center 2013-04-106-001). The ethics committee granted a waiver of consent for this retrospective chart review study.

### Data collection

Demographic, clinical, and hospital data were obtained by reviewing patient medical records. All operations were performed by two pediatric cardiac surgeons. General anesthesia was induced with thiopental and midazolam and maintained with isoflurane. Tracheal intubation was facilitated with rocuronium. During the CPB period, midazolam, fentanyl, and rocuronium were infused via CPB circuit to maintain anesthesia. Cardiac arrest was induced under CPB and aortic cross clamp using crystalloid or blood cardioplegia. Histidine-tryptophan-ketoglutarate (HTK) solution (Custodiol, Alsbach, Germany) was used as a crystalloid cardioplegia. Blood cardioplegia was provided by a mixture of packed red blood cells and a commercially available crystalloid solution (JW Pharmaceutical, Seoul, Korea) at a ratio of 4:1. The initial cardioplegic solution (30 mL/kg) was infused at a pressure of 50–60 mmHg. Additional solution (15 mL/kg) was infused every 20 min in cases of blood cardioplegia. Hemodynamic monitoring included electrocardiography, pulse oximetry, esophageal-rectal temperature, invasive radial artery pressure monitoring, and central venous pressure. Arterial blood gas and electrolytes were measured at baseline, during the rewarming period, and every 30 min thereafter. Modified ultrafiltration was performed after CPB weaning.

Pediatric Risk of Mortality (PRISM III) scores were calculated with age-related physiological parameters including systolic blood pressure, heart rate, temperature, pupillary reflexes, mental status, acidosis (pH and total CO_2_), pCO_2_, pO_2_, glucose, potassium, creatinine, blood urea, white blood cell counts, platelet counts, and prothrombin or partial thromboplastin times (Pollack et al. 1988). This scoring system has a range of 0–74, and a higher score indicates a higher mortality risk.

We determined in-hospital outcome variables by review of medical records. The postoperative morbidity rates, such as neurologic complications, arrhythmia, pulmonary congestion, pneumonia, jaundice, or acute kidney injury were determined. We attempted to identify neurologic complications including seizures, coma, cerebral edema, or cerebral lesions documented on imaging studies included in the medical records. Acute kidney injury was defined as a ≥50 % increase in serum creatinine or an absolute increase in serum creatinine of ≥0.3 mg/dL. We also determined the lengths of postoperative hospital stay (intensive care unit and total hospital days) as an additional outcome variable.

### Statistical analysis

All statistical analyses were performed with the SPSS software package (version 19.0; SPSS Inc, Chicago, IL). Continuous clinical variables are presented as means and SDs or as medians and ranges. The patients were classified by the following clinical definitions: hyponatremia prior to CPB (serum sodium ≤135 mmol/L), severe hyponatremia during CPB, (serum sodium ≤130 mmol/L), and hypernatremia after CPB (serum sodium ≥145 mmol/L). Comparisons between two groups were conducted using an unpaired *t*-test or the Mann–Whitney U-test, as appropriate. Distributions of categorical variables among groups were assessed using a Chi-square test. Generalized linear models were used to find independent prognostic factors. Two-sided p-values <0.05 were considered statistically significant.

## Results

The median patient age (145 male and 130 female) was 14 months (range 1–180 months) and the median PRISM score was 2 (range 0–10) (Table 1). The mean duration of operation and CPB were 203 ± 36 and 69 ± 22 min, respectively (Table 2).

**Table 1:** Baseline patient characteristics (n = 275)

Age, months [median (range)]	14 (1–180)
Male/female, n (%)	145/130 (52.7/47.3 %)
Weight, kg [median (range)]	9.6 (2.4–83)
Body surface area, m^2^ [median (range)]	0.46 (0.18–2.03)
PRISM score [median (range)]	2 (0–10)
Serum creatinine, mg/dL (mean ± SD)	0.38 ± 0.14

*PRISM* pediatric risk of mortality

**Table 2:** Cardio-pulmonary bypass procedure

Operation duration, min (mean ± SD)	203 ± 36
CPB duration, min (mean ± SD)	69 ± 22
Priming volume, mL (mean ± SD)	124 ± 65
Serum sodium, mmol/L (mean ± SD)	
Prior to CPB	139.3 ± 3.0
Lowest during CPB	135.7 ± 4.3
After the end of CPB	142.7 ± 4.0
Serum glucose, mg/dL (mean ± SD)	157 ± 44
Nasopharyngeal temp, ºC (mean ± SD)	31.0 ± 1.8

*CPB* cardiopulmonary bypass

**Fig. 1:**
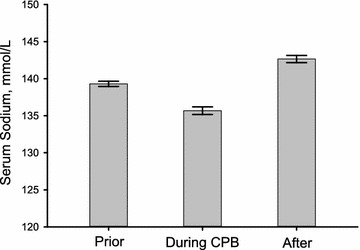
Acute serum sodium concentration changes during cardiopulmonary bypass (*error bar*; mean concentration with 95 % CI)

Changes in serum sodium concentrations during CPB are shown in Fig. 1. Prior to heart surgery, hyponatremia (≤135 mmol/L) was observed in 21 of 275 patients. After initiation of CPB, serum sodium decreased significantly (*P* < 0.001) and severe hyponatermia (≤130 mmol/L) developed in 32 patients. At the end of CPB, however, serum sodium concentrations immediately increased (*P* < 0.001) above preoperative levels and hypernatremia (≥145 mmol/L) rather than hyponatremia developed in 86 patients.

Postoperative complications developed in 70 of the 275 patients. Among those with complications, 25 patients suffered two or more complications. Acute kidney injury was the most common complication and occurred in 38 patients. Pulmonary congestion and pneumonia developed in 19 and 21 patients, respectively. Additionally, arrhythmias developed in 8 patients and liver dysfunction developed in 5 patients. Neurologic complications were rare and transient ankle jitteriness or headache developed in 3 patients.

Associations between serum sodium concentrations and in-hospital outcomes are shown in Table 3. The patients with preoperative hyponatremia had longer hospital stays (intensive care unit and total hospital days) and higher postoperative complication rates. However, the degree of acute serum sodium changes during CPB was not associated with poor patient outcomes. On the other hand, increased serum sodium concentrations at the end of CPB were associated with longer hospital stays and higher morbidity rates.

**Table 3:** Serum sodium concentrations and postoperative outcomes

Outcomes	Prior to CPB (mmol/L)			Lowest during CPB (mmol/L)			After CPB (mmol/L)		
	≤135	>135	*P*	≤130	>130	*P*	≤145	>145	*P*
ICU morbidity, events [n (%)]	12/21 (57 %)	58/254 (23 %)	0.001	4/32 (13 %)	66/243 (27 %)	0.074	31/189 (16 %)	39/86 (45 %)	<0.001
Length of ICU stay, days [median (range)]	5 (3–10)	2 (1–13)	<0.001	2 (2–9)	2 (1–13)	0.057	2 (1–10)	3.5 (2–13)	<0.001
Length of hospital stay, days [median (range)]	9 (7–22)	7 (5–21)	<0.001	7 (5–13)	7 (5–22)	0.900	7 (5–19)	8.5 (6–22)	<0.001

*CPB* cardiopulmonary bypass, *ICU* intensive care unit

Independent prognostic factors for patient outcomes were assessed using generalized linear models (Table 4). Lower serum sodium concentrations prior to and higher serum sodium concentrations after CPB, along with younger age and longer operation times, were independently associated with poor in-hospital outcomes.

**Table 4:** Generalized linear models predicting postoperative outcomes

Co-variables	ICU morbidity			Length of ICU stay (day)^a^		
	Coefficient (*B*)	Standard error of *B*	*P*	Coefficient (*B*)	Standard error of *B*	*P*
Age (months)	−2.548	0.4285	<0.001	−0.213	0.0199	<0.001
Gender (male)	0.393	0.3867	0.310	0.004	0.0193	0.844
PRISM III (score)	−0.178	0.1021	0.080	0.002	0.0050	0.660
Operation duration (min)	0.019	0.0060	0.001	0.001	0.0003	<0.001
Serum sodium (mmol/L)						
Prior to CPB	−0.206	0.0808	0.011	−0.010	0.0039	0.013
Lowest during CPB	−0.010	0.0558	0.857	−0.001	0.0028	0.622
After the end of CPB	0.133	0.0614	0.031	0.007	0.0031	0.019

*CPB* cardiopulmonary bypass, *ICU* intensive care unit

^a^Log-transformed values were used for the analyses

## Discussion

In this observational study, serum sodium concentrations substantially decreased after initaiation of CPB, and then immediately increased above preoperative levels at the completion of CPB. Acute and transient hyponatremia during CPB was not associated with patient outcomes. However, preoperative hyponatremia and overcorrection of serum sodium at the end of CBP were associated with longer hospital admission lengths and higher morbidity rates.

The clinical implication of sodium disturbances during CPB has not been well documented. In this study, serum sodium concentrations significantly decreased, and severe hyponatremia, defined as a serum sodium concentration less than 130 mEq/L, was frequently observed during CPB. Possible mechanisms for hyponatremia are likely multifactorial, but include etiologies such as fluid overload (prime solutions, cardioplegia, and transfusions) (Darling et al. 2000), diuretic administration, impaired water excretion caused by the release of anti-diuretic hormone (Philbin et al. 1977), and CPB-related natriuretic effect (Sehested et al. 1997). Theoretically, hyponatremia during CPB may contribute to cerebral edema or related complications if associated with a decrease in serum osmolality. However, we did not have sufficient data on temporal osmolality changes during CPB in this study, and the degree of acute serum sodium decrease during CPB was not associated with patient outcomes. A recent study conducted in cardiac surgery patients showed that acute hyponatremia during cardioplegia with HTK solution was isotonic and that the measured osmolality did not change during surgery (Lindner et al. 2012). If acute decreases in serum sodium during CPB was due to pseudohyponatremia with a normal plasma tonicity, then harmful effects would not likely result.

In previous studies regarding baseline serum sodium concentrations, hyponatremia has been established as a poor prognostic factor and may predict poor outcomes or longer hospital stay in patients with congestive heart failure (Mohammed et al. 2010). Hyponatremia was also an independent risk factor of poor outcomes in critically ill patients admitted to the ICU (Stelfox et al. 2008). Heart surgery patients may be critically ill and have comorbidities such as heart failure. A number of clinical and surgical factors have been identified as predictors of poor outcomes after heart surgery, including heart failure, kidney injury, comorbidities, and prolonged or complicated surgical procedures. Hyponatremia is commonly associated with those factors, and a recent study has shown that baseline hyponatremia is an independent risk factor for adverse events, prolonged hospital stay, and mortality after heart surgery (Lindner et al. 2012). Our results may also provide additional information regarding the relationships between preoperative hyponatremia and in-hospital outcomes in pediatric patients undergoing heart surgery with CPB.

At the conclusion of CPB, hypernatremia (≥145 mmol/L) was observed in approximately one-third of patients and was associated with longer hospital stays and higher morbidity rates. The prevalence of postoperative hypernatremia in patients after cardiac surgery has rarely been demonstrated, and the impact on clinical outcomes has been unclear. Hypernatremia has only recently been shown to be a common event in intensive care units after major cardiothoracic surgery, and was associated with an increased risk of hospital mortality (Lindner et al. 2010). However, the cause of hypernatremia after cardiac surgery is not clear. On the review of medical and surgical records in this study, there were no administrations of hypertonic saline by the anesthesiologist; however, sodium bicarbonate was frequently administered during CPB to correct for metabolic acidosis or acute hyponatremia. Unlike hyponatremia, which would be iso- or even hyperosmotic, hypernatremia is consistently associated with hyperosmolality. The overcorrection of hyponatremia, which might have been iso-osmotic during CPB (Lindner et al. 2012) or partially compensated for by adaptation mechanisms (Verbalis and Gullans 1991; Gullans and Verbalis 1993), may result in cell shrinkage, which might cause various complications and poor outcomes. For example, cerebral shrinkage can lead to neurologic impairment. Moreover, a decrease in cardiac contractility (Lenz et al. 1986; Kozeny et al. 1985) and disturbances in glucose metabolism (Bratusch-Marrain and DeFronzo 1983) and immunologic functions (Kuroda et al. 1997) have been described in association with hypernatremic hyperosmolar states. Thus, overcorrection of hyponatremia should be viewed as an emergent problem and avoided in patients undergoing CPB.

This study has several limitations. First, data on osmolality was not available, and thus we could not distinguish between hypoosmolar, isoosmolar, and hyperosmolar hyponatremia. Second, our study was retrospective and observational in nature. As such, our observations are valuable for generating hypotheses but not causal inference. Third, the sample size was small, resulting in limited power to detect associations by statistical analyses. Specifically, all possible confounders could not be concurrently introduced in the multivariable analysis. Forth, this study included patients undergoing repair of ventricular or atrial septal defects at a single center (to minimize the effects of confounding factors on patient outcomes), which makes drawing generalized conclusions more difficult. It is possible that patients undergoing other types of cardiac surgery or other patient populations may have different experiences.

In conclusion, serum sodium concentrations substantially decreased after initaiation of CPB, and then immediately increased above preoperative levels at the end of CPB. Acute and transient hyponatremia during CPB was not related to patient outcomes. However, overcorrection of serum sodium at the conclusion of CPB was associated with poor in-hospital outcomes. These results suggest that acute hyponatremia during CPB does not warrant immediate treatment by raising serum sodium levels, and caution is required to avoid serum sodium overcorrection on the conclusion of CPB.
